# Wearable Device–Based Intervention for Promoting Patient Physical Activity After Lung Cancer Surgery: A Nonrandomized Clinical Trial

**DOI:** 10.1001/jamanetworkopen.2024.34180

**Published:** 2024-09-20

**Authors:** Junghee Lee, Sunga Kong, Sumin Shin, Genehee Lee, Hong Kwan Kim, Young Mog Shim, Juhee Cho, Danbee Kang, Hye Yun Park

**Affiliations:** 1 Department of Thoracic and Cardiovascular Surgery, Samsung Medical Center, Sungkyunkwan University School of Medicine, Seoul, South Korea; 2 Department of Clinical Research Design and Evaluation, Samsung Advanced Institute for Health Sciences and Technology, Sungkyunkwan University, Seoul, South Korea; 3 Patient-Centered Outcomes Research Institute, Samsung Medical Center, Seoul, South Korea; 4 Department of Thoracic and Cardiovascular Surgery, College of Medicine, Ewha Womans University Mokdong Hospital, Seoul, South Korea; 5 Trend Sensing and Risk Modeling Center, Institution of Quality of Life in Cancer, Samsung Medical Center, Seoul, South Korea; 6 Center for Clinical Epidemiology, Samsung Medical Center, Sungkyunkwan University School of Medicine, Seoul, South Korea; 7 Department of Epidemiology and Medicine, Welch Center for Prevention, Epidemiology, and Clinical Research, Johns Hopkins Bloomberg School of Public Health, Baltimore, Maryland; 8 Division of Pulmonary and Critical Care Medicine, Department of Medicine, Samsung Medical Center, Sungkyunkwan University School of Medicine, Seoul, South Korea

## Abstract

**Importance:**

Emerging evidence suggests that wearable devices are feasible for monitoring physical activity among patients with lung cancer. However, the association between wearable devices and improvement in patient recovery after surgery remains underexplored.

**Objective:**

To evaluate the effects of a wearable device intervention on the recovery of physical activity, cardiopulmonary function, and health-related quality of life (HRQOL) after lung cancer surgery.

**Design, Setting, and Participants:**

This nonrandomized clinical trial with a historical control was conducted at a single tertiary cancer center (Samsung Comprehensive Cancer Center) in Seoul, South Korea, between October 18, 2018, and May 24, 2019. Patients were included if they had suspected or confirmed non–small cell lung cancer scheduled for curative surgery more extensive than lobectomy and had an Eastern Cooperative Oncology Group status of 0 or 1. Patients were compared with historical control participants from data collected between September 20, 2017, and September 10, 2018, as part of the Coordinated Approach to Cancer Patients’ Health for Lung Cancer (CATCH-LUNG) prospective cohort study. Data analysis was performed between June 21 and July 16, 2020.

**Intervention:**

A personalized exercise regimen monitored via a wearable device was administered to intervention patients at home in 3 stages: preoperative (from diagnosis to surgery), immediate (from discharge to 2 months after surgery), and later postoperative (from 2 to 6 months after surgery). Control patients received usual care.

**Main Outcomes and Measures:**

The primary outcome was cardiopulmonary function, and the co–primary outcome was physical activity at 6 months after surgery, measured with 6-minute walking distance (6MWD) and number of daily steps, using a linear regression model. Secondary outcomes were changes in cardiopulmonary function, physical activity, and HRQOL, including function and symptoms from baseline to 2 weeks and 6 months after surgery. Additionally, cardiopulmonary function and physical activity (number of daily steps and time spent on moderate-to-vigorous physical activity [MVPA]) at 2 weeks after surgery, physical activity (time spent on MVPA) at 6 months after surgery, and HRQOL, including function and symptoms at 2 weeks and 6 months after surgery, were assessed as secondary outcomes.

**Results:**

This trial included 74 patients in the intervention group (mean [SD] age, 60.4 [8.7] years; 31 [41.9%] men and 43 [58.1%] women) and 120 in the control group (mean [SD] age, 60.2 [8.7] years; 65 [54.2%] men and 55 [45.8%] women). Daily steps, MVPA, and 6MWD decreased initially at 2 weeks after surgery but increased thereafter. The control group had a larger decrease in the number of daily steps from baseline compared with the intervention group (−4877 [95% CI, −5861 to −3893] steps vs −1753 [95% CI, −2968 to −539] steps) at 2 weeks after surgery. By 6 months after surgery, the intervention group increased their daily steps by 2220 (95% CI, 1006 to 3435) from baseline, whereas the control group did not return to their baseline number of steps. The intervention group had significantly more daily steps (12 321 [95% CI, 8749-15 761] vs 10 118 [95% CI, 7341-13 420]; *P* = .007) and had greater vigorous physical activity (33.6 [95% CI, 13.5 to 59.8] vs 18.5 [5.7 to 40.8] minutes; *P* = .003) at 6 months after surgery compared with the control group. No difference in 6MWD was found. However, the intervention group had better patient-reported physical function (mean [SD] score, 82.2 [17.3] vs 76.9 [17.5]; *P* = .04), less dyspnea (mean [SD] score, 24.8 [27.1] vs 34.5 [31.6]; *P* = .03), and less pain (mean [SD] score, 21.4 [20.2] vs 30.1 [26.8]; *P* = .01) at 2 weeks after surgery and less dyspnea (mean [SD] score, 5.4[12.4] vs 12[23.3]; *P* = .01) at 6 months after surgery compared with the control group.

**Conclusions and Relevance:**

In this nonrandomized clinical trial, integration of perioperative exercise interventions using wearable devices improved physical activity (especially MVPA) and dyspnea at 6 months after lung cancer surgery compared with usual care. This finding suggests a promising role for wearable devices in personalizing perioperative rehabilitation strategies.

**Trial Registration:**

ClinicalTrials.gov Identifier: NCT03215537

## Key Points

**Question:**

Is a wearable-based intervention effective for improving physical activity, cardiopulmonary function, and patient-reported outcomes after lung cancer surgery?

**Findings:**

In this nonrandomized clinical trial with 74 intervention patients and 120 control patients, the intervention group had a smaller decrease in number of daily steps and time spent on moderate-to-vigorous physical activity (MVPA) at 2 weeks after surgery and a larger increase in daily steps and MVPA at 6 months after surgery.

**Meaning:**

The wearable device intervention used in this trial improved physical activity and patient-reported dyspnea at 6 months after surgery, and these findings may guide the development of comprehensive rehabilitation programs that integrate wearable devices.

## Teaser

This nonrandomized clinical trial examines the effects of a wearable-based intervention on physical activity, recovery of cardiopulmonary function, and patient-reported outcomes after lung cancer surgery.

## Introduction

Surgery is the primary treatment for early-stage non–small cell lung cancer (NSCLC). The 5-year estimated survival rate for stage I NSCLC after curative resection is greater than 70%.^1^ However, most patients experience a substantial reduction in pulmonary function, especially in the immediate postoperative period.^2^ This decreased lung function is correlated with decreased physical activity, cardiopulmonary function, and health-related quality of life (HRQOL) including patient-reported physical function and postoperative symptoms.^3,4^ Cardiopulmonary function generally improves over time as lung function increases, but 24% of patients do not return to their prior cardiopulmonary capacity at 6 months after surgical resection.^3^ In particular, the duration and intensity of physical activity at 2 weeks after surgery, as well as baseline physical activity, is predictive of recovery of long-term cardiopulmonary function. Thus, interventions to enhance perioperative physical activity (eg, aerobic exercise) are necessary after lung cancer surgery.^5^

In a 2022 systematic review that included 10 randomized clinical trials, preoperative exercise programs resulted in a significant reduction (by as much as 55%) in the risk of developing pulmonary complications and an average decrease of 2 days in hospital stay.^6^ Preoperative exercise training led to increased postoperative physical performance, as measured by maximal oxygen consumption; however, its effects on physical performance, as measured by the 6-minute walk test (6MWT), were uncertain.^6^ Postoperative exercise is also known to promote the recovery of cardiopulmonary function and to improve the physical component of HRQOL and symptoms.^7^ Despite the effectiveness of perioperative exercise training in patients with lung cancer, most studies were conducted under strict supervision and focused on the effects of breathing exercises and on-site exercise programs.^6,7^ This supervised training often limits patient access to facilities due to inflexible usage schedules and long distances from home. Thus, clinical evidence has not been translated into daily life, and most patients often do not meet the recommended physical activity levels.^8^ Accordingly, interest is growing regarding the use of nonsupervised and home-based training to promote and maintain physical activity.

Over the past decade, electronic health devices, such as wearable activity trackers, have emerged as promising tools for monitoring patient activity. Recent studies have reported on the safety and feasibility of these devices for patients using them after gynecologic and abdominal surgeries.^9^ In addition, unsupervised monitoring with wearable devices has been used with patients with cancer. A 2022 systematic review provided preliminary evidence on the efficacy of these devices in promoting long-term physical activity among patients with cancer.^10^

Patients with lung cancer tend to be older^11^ and are more likely to have respiratory symptoms and limited pulmonary function due to the nature of the disease, which can be a barrier to application of unsupervised exercise programs in this population. Recently, accumulated data from wearable devices demonstrated their capability to monitor patient activity intensity, time, frequency, and volume, which are crucial for modifying exercise programs to ensure safety and efficacy.^12,13^ Several studies with a small number of participants also demonstrated the feasibility of wearable devices in patients with lung cancer.^12,13,14^

To our knowledge, no trial has yet investigated whether wearable-based interventions may improve physical activity, cardiopulmonary function, and patient-reported HRQOL after lung cancer surgery. Therefore, we conducted the present trial to evaluate the efficacy of wearable device use, compared with usual care, before and up to 6 months after surgery in promoting physical activity and cardiopulmonary function while decreasing symptoms in patients with NSCLC after surgery.

## Methods

The Samsung Medical Center Institutional Review Board reviewed and approved this nonrandomized clinical trial. The trial protocol and statistical analysis plan are presented in Supplement 1. All study participants provided written informed consent. The study followed the Transparent Reporting of Evaluations With Nonrandomized Designs (TREND) reporting guideline.

### Trial Design and Participants

This nonrandomized clinical trial with a historical control was conducted at a single tertiary cancer center (Samsung Comprehensive Cancer Center) in Seoul, South Korea, between October 18, 2018, and May 24, 2019.^15^ We selected a control group from the Coordinated Approach to Cancer Patients’ Health for Lung Cancer (CATCH-LUNG) prospective cohort study,^16^ which had the same study site and same trial protocol except for the intervention. Patients in the intervention group may have stayed in the same general ward as those in the control group; to reduce bias, we divided the study into period 1 (before intervention for the historical control group) and period 2 (intervention period for the intervention group) (Supplement 1), and we selected a control group from the same setting for a year before intervention to prevent time effects.

The inclusion criteria for the intervention group were as follows: (1) suspected or histologically confirmed NSCLC scheduled for curative surgery more extensive than lobectomy, including bilobectomy and pneumonectomy; (2) Eastern Cooperative Oncology Group performance status of 0 or 1; (3) no walking problems; and (4) understanding of the purpose of the study and consent to participate.

We excluded patients if they met any of the following criteria: (1) had multiple primary cancers, metastatic lung cancer, recurrent lung cancer, or a history of other cancers in the past 3 years; (2) had received neoadjuvant chemotherapy, radiotherapy, or both; (3) did not have baseline variables due to changes in the surgical plan, treatment plan, or withdrawal of consent; (4) were pathologically confirmed after surgery not to have NSCLC; and (5) had limited ability to participate in the intervention due to heart failure (New York Heart Association functional classes III and IV), cerebrovascular disease (eg, cerebral hemorrhage, cerebral infarction, transient ischemic attack, stroke, or cerebrovascular accident), walking impairment (eg, arthritis, osteoporosis, joint pain, or rheumatoid arthritis), or kidney disease (eg, chronic kidney failure).

Control participants were selected in accordance with Drug Information Association Adaptive Design Scientific Working Group guidelines,^17^ using data collected from September 20, 2017, and September 10, 2018, as part of the CATCH-LUNG prospective cohort study. The protocols for CATCH-LUNG patient enrollment and data collection were described in a previous study.^16^ The inclusion and exclusion criteria were applied in line with the aforementioned criteria for the intervention group (eFigure 1 in Supplement 2).

Due to the nature of the intervention, patients were not randomly assigned. In addition, patients and investigators were not blinded during the study. However, the data analysts and investigators who observed patient outcomes during follow-up were blinded to minimize bias.

### Study Groups

#### Intervention Group

The intervention was designed to include 3 phases: preoperative intervention (from diagnosis to surgery), immediate postoperative intervention (from discharge to 2 months after surgery), and postoperative intervention (from 2 to 6 months after surgery). In the intervention group, the initial exercise program consisted of 30 minutes of general education about exercise.

A cardiopulmonary exercise test was performed before training to determine the optimal individual training dose and to rule out cardiopulmonary limitations or contraindications. The cardiopulmonary exercise test was a mandatory element of our web-based approach to prescribe adequate exercise and protect participants from overload. If the participant had reached the target set for the previous week (>70% completion rate), the target was reset. The exercise program was personalized based on the fitness test performed at baseline according to American College of Sports Medicine guidelines.^18^ The routine included a 5-minute warm-up, 30 to 90 minutes of endurance training (starting at 45%-70% of heart rate reserve), respiratory muscle training with an inspirometer (10 repetitions, 3 times per day), and a 5-minute cool-down, performed 5 days per week. In addition, patients were given a guidebook on how to perform the muscle strength exercises. All exercises were clearly explained and demonstrated at the beginning of the 3 phases (preoperative intervention, immediate postoperative, and postoperative intervention), and patients were asked to perform the program at home and unsupervised. Throughout the intervention period, a wearable device (Fitbit Versa, version 1 [Fitbit Inc] [hereinafter, device A]) was used to monitor step count, activity intensity, activity time, frequency, and heart rate during exercise to provide real-time feedback. Using the monitored patient activity data, an exercise physiologist with 20 years of experience (S.K.) conducted transtheoretical model–based patient counseling through weekly or biweekly telephone calls, using web-based data from the intervention device. Physical activity levels were promoted by setting goals for gradual increases in number of daily steps and time spent on moderate-to-vigorous physical activity (MVPA).^19,20^ However, participants were not provided with incentives to improve compliance or adherence.

#### Control Group

The control group received only usual care, which comprised inspiratory exercises using an incentive spirometer and walking exercises. No specific individual guidance on exercise was provided to the control group, particularly regarding preoperative and postoperative activities.

### Follow-Up and Outcome Variable Definitions

After baseline measurements, we followed up patients at 2 weeks and 6 months after surgery to measure outcomes. The primary outcome was cardiopulmonary function, and the co–primary outcome was physical activity at 6 months after surgery, measured with 6-minute walking distance (6MWD) and number of daily steps. Physical activity was also measured with time spent on MVPA as a secondary outcome. Secondary outcomes were changes in cardiopulmonary function, physical activity, and HRQOL, including function and symptoms from baseline to 2 weeks and 6 months after surgery. Additionally, cardiopulmonary function and physical activity (number of daily steps and time spent on MVPA) at 2 weeks after surgery, physical activity (time spent on MVPA) at 6 months after surgery, and HRQOL, including function and symptoms at 2 weeks and 6 months after surgery, were assessed as secondary outcomes.

Physical activity was assessed using a reliable wearable device at baseline (Fitbit Flex, version 1 [hereinafter, device B] for both groups), at 2 weeks after surgery (device B for the control group and device A for the intervention group), and at 6 months after surgery (device B for both groups). Device B quantified steps per day and MVPA time by intensity; it was applied for 7 consecutive days to measure the physical activity level during weekdays and weekends before the visit. Additionally, it was worn on the wrist without a screen on the device; thus, patients could not monitor their physical activity level. Per the CATCH-LUNG protocol for the prospective cohort, physical activity in the control group was assessed with device B at baseline, 2 weeks after surgery, and 6 months after surgery. Physical activity in the intervention group was assessed with device B at baseline and at 6 months after surgery; physical activity at 2 weeks after surgery could not be accessed with device B due to the use of device A throughout the intervention period. Thus, data on physical activity at 2 weeks after surgery in the intervention group were assessed using device A because it had been used in the intervention program.

Cardiopulmonary function was tested using the 6MWT according to American Thoracic Society guidelines.^21^ Each participant was asked to walk (not run) back and forth along the corridor as briskly as possible so that the longest possible distance was covered in 6 minutes.

Participant HRQOL including function and symptoms was evaluated using a validated Korean version of the European Organisation for Research and Treatment of Cancer 30-Item Core Quality of Life Questionnaire (EORTC QLQ-C30).^22^ The EORTC QLQ-C30 is composed of 5 functional scales (physical, social, role, emotional, and cognitive functioning), symptom scales (dyspnea, pain, fatigue, nausea and vomiting, insomnia, appetite loss, constipation, and diarrhea), and a global health status and quality-of-life scale. The EORTC QLQ-C30 items were scored according to the scoring manual, and the data were linearly transformed to yield scores ranging from 0 to 100. Higher scores indicated a worse status in the symptom domains. Mean differences of 10 points or more are widely viewed as clinically significant when interpreting the results of the QLQ-C30.^23^

Patient information, including age, sex, comorbidities, and smoking status, was collected before surgery. Outcomes, including physical activity using the wearable device, cardiopulmonary function, and patient-reported HRQOL (including function and symptoms), were measured before surgery and at 2 weeks (median, 2 weeks; range, 1-7 weeks) and 6 months (median, 5 months; range, 3-9 months) after surgery. Clinical information, including type of surgical approach, pathologic type of lung cancer, pathologic stage, and adjuvant treatment, was also collected from the electronic medical record after surgery. Postoperative pulmonary complications were prospectively collected according to agreed-upon definitions.

### Statistical Analysis

Given that we had co–primary end points, our sample size calculation was based on the primary end point that necessitated the larger sample size of the 2. In this study, we aimed to compare 6MWD and physical activity at 6 months after surgery between the intervention and control groups, with a mean effect size (Cohen *d* = 0.5) of α = .05 and 90% power. To achieve this, we needed 85 participants per group. Allowing for 20% loss to follow-up, we needed 100 participants per group.

We used an intention-to-treat approach for all analyses. Outcomes were compared using linear regression and a mixed model at each time point and change, respectively. For outcomes measured at 3 visits, we used linear mixed-effects models with the main effects of visits and visit group interaction. Random intercept effects were included to account for differences in outcomes among study participants at baseline.

A sensitivity analysis was conducted using a propensity score (PS)-matched population to allow for a more accurate comparison between the 2 groups. The logistic regression model included all covariates to estimate the probability of receiving treatment based on their respective covariates. Additionally, season and social function at baseline were included as covariates in the logistic regression model. To reduce bias, we implemented 1:1 nearest-neighbor matching using the PS with a caliper of 0.2 on the score scale.^24^ Statistical significance was set at *P* <.05 (2-tailed). All analyses were performed between June 21 and July 16, 2020, using Stata, version 16 (StataCorp LLC).

## Results

This study included 194 patients: 74 in the intervention group (mean [SD] age, 60.4 [8.7] years; 31 [41.9%] men and 43 [58.1%] women) and 120 in the control group (mean [SD] age, 60.2 [8.7] years; 65 [54.2%] men and 55 [45.8%] women) (Table 1). A total of 104 patients were enrolled initially but 1 was excluded due to cancellation of the operation and 3 patients refused to participate in outcome measurements after enrollment. Of the 100 patients randomized, 7 patients were unable to receive the intervention. Of the 93 patients who received the intervention, 19 were lost to follow-up because they were receiving chemotherapy (eFigure 2 in Supplement 2). In the CATCH-LUNG cohort, which served as the historical control group, 169 patients were enrolled initially but 10 refused to participate. Therefore, 159 patients were observed, with 39 lost to follow-up, leaving 120 control patients for analysis (eFigure 2 in Supplement 2). Patients lost to follow-up were more likely to be male and to have a lower level of education (eTable 1 in Supplement 2). However, there were no significant differences in other clinical characteristics between the patients who were lost to follow-up and those who were not. Among the participants, there was a 95% compliance rate with the use of wearable devices. The proportion of lobectomy was similar between the 2 groups (97.3% in the intervention group and 94.2% in the control group), as was the rate of postoperative pulmonary complications (5.4% in the intervention group and 6.7% in the control group).

**Table 1.** Patient Characteristics at Baseline^a^

Characteristic	Patient group		*P* value
	Intervention (n = 74)	Control (n = 120)	
Age, mean (SD), y	60.4 (8.7)	60.2 (8.7)	.86
Sex			
Male	31 (41.9)	65 (54.2)	.10
Female	43 (58.1)	55 (45.8)	
BMI, mean (SD)	23.9 (3.2)	24.2 (2.9)	.47
Body fat, mean (SD), %	28.5 (7.0)	27.5 (7.6)	.36
Muscle mass, mean (SD), kg	24.8 (5.4)	25.8 (5.4)	.19
Married	60 (81.1)	99 (82.5)	.80
Education			
Less than high school	16 (21.6)	28 (23.3)	.78
High school or higher	58 (78.4)	92 (76.7)	
Employment status			
Unemployed	29 (39.2)	54 (45.0)	.43
Employed	45 (60.8)	66 (55.0)	
Income (million Korean won)	400 (300-700)	400 (210-500)	.64
Smoking status			
Never	44 (59.5)	59 (49.2)	.31
Past	24 (32.4)	45 (37.5)	
Current	6 (8.1)	16 (13.3)	
Religious affiliation			
Yes	29 (39.2)	49 (40.8)	.82
No	45 (60.8)	71 (59.2)	
Comorbidity			
Cardiac	4 (5.4)	12 (10.0)	.26
Pulmonary	8 (10.8)	24 (20.0)	.09
Thrombosis	0 (0.0)	3 (2.5)	.29
Hypertension	23 (31.1)	34 (28.3)	.68
Diabetes	5 (6.8)	16 (13.3)	.15
Cerebrovascular accident	0	3 (2.5)	.29
Pulmonary function test			
FVC, L	3.4 (3.0-4.1)	3.4 (3.0-4.3)	.67
FVC, %	96 (89-105)	95 (88-105)	.14
FEV_1_, L	2.6 (2.3-3.0)	2.6 (2.3-3.1)	.48
FEV_1_, %	94 (87-107)	94 (87-103)	.31
FEV_1_/FVC, %	76 (71-80)	76 (71-79)	.94

Abbreviations: BMI, body mass index (calculated as weight in kilograms divided by height in meters squared); FEV_1_, forced expiratory volume in 1 second; FVC, forced vital capacity.

^a^ Unless indicated otherwise, values are presented as No. (%) of patients.

After matching, there were 57 patients each in the control and intervention groups. Characteristics, including season at enrollment, were well balanced (eTable 2 in Supplement 2).

For the intervention and control groups, 6MWD at 6 months after surgery was not significantly different. However, the number of daily steps at 6 months after surgery was significantly higher in the intervention group than in the control group (12 321 [95% CI, 8749 to 15 761] vs 10 118 [95% CI, 7341 to 13 420]; *P* = .007; Table 2).

**Table 2.** Six-Minute Walking Distance and Physical Activity at Baseline and 2 Weeks and 6 Months After Surgery, by Group

Group	Baseline		2 wk After surgery		6 mo After surgery	
	Mean (95% CI)	*P* value	Mean (95% CI)	*P* value	Mean (95% CI)	*P* value
**6-min walking distance, m**						
Control	511 (463 to 574)	.05	450 (401 to 510)	.19	513 (480 to 555)	.12
Intervention	539 (510 to 575)		468 (420 to 504)		536 (495 to 564)	
**No. of daily steps**						
Control	10 254 (7176 to 13 512)	.14	5683 (3570 to 8900)	<.001	10 118 (7341 to 13 420)	.007
Intervention	9411 (6652 to 11 398)		8267 (6295 to 11 043)		12 321 (8749 to 15 761)	
**Level of physical activity, min**						
Vigorous						
Control	18.5 (5.9 to 37.5)	.66	6.2 (0.3 to 16.9)	<.001	18.5 (5.7 to 40.8)	.003
Intervention	16.3 (5.1 to 33.8)		17.1 (6.2 to 37.7)		33.6 (13.5 to 59.8)	
Moderate						
Control	19.4 (9.2 to 37.2)	.43	10.1 (2.4 to 23.3)	<.001	19.8 (9.7 to 35.0)	.24
Intervention	17.4 (8.6 to 27.2)		21.2 (10.2 to 33.4)		23.9 (12.7 to 34.8)	
Light						
Control	243.6 (183.6 to 296.1)	.90	144.8 (94.8 to 196.4)	.009	240.6 (191.1 to 282.7)	.89
Intervention	233.5 (180.0 to 288.3)		163.8 (131.3 to 206.3)		236.4 (182.7 to 289.4)	

**Figure 1.**
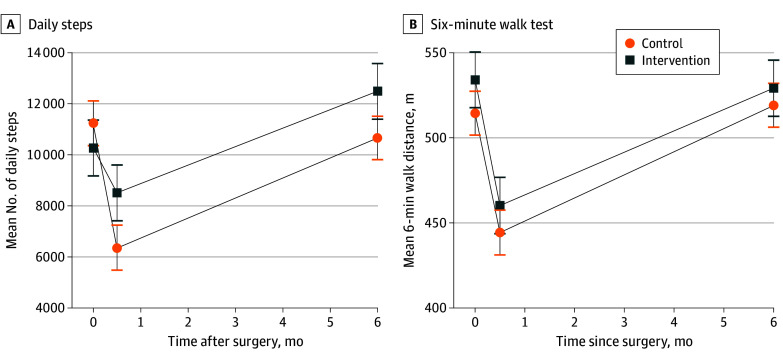
Changes in Daily Steps and 6-Minute Walking Distance by Group The number of daily steps (A) and 6-minute walking distance in meters (B) are reported as means (95% CIs). Outcomes were measured at baseline (before surgery), 2 weeks after surgery, and 6 months after surgery.

Two weeks after surgery, the number of daily steps decreased by −4877 (95% CI, −5861 to −3893) and −1753 (95% CI −2968 to −539) from baseline in the control and intervention groups, respectively (Figure 1A). At 6 months after surgery, the intervention group had an increase in daily steps (2220 [95% CI, 1006 to 3435]), whereas the control group still did not return to the baseline number of daily steps (−586 [95% CI, −1551 to 379]). Time spent on physical activity at 2 weeks after surgery was higher in the intervention group than in the control group. Particularly, time spent on vigorous physical activity at 6 months after surgery was significantly higher in the intervention group than in the control group (33.6 [95% CI, 13.5 to 59.8] vs 18.5 [95% CI, 5.7 to 40.8] minutes; *P* = .003; Table 2). Differences between groups in changes in 6MWD 6 months after surgery were not significant (Figure 1B and Table 2). In the matched population, 6MWD at 6 months after surgery was significantly higher in the intervention group (mean, 536.0 [95% CI, 495.0 to 571.0] m) than in the control group (510.0 [95% CI, 480.0 to 540.0] m) (*P* = .02). Number of daily steps was also significantly higher in the intervention group (12 717 [95% CI, 9582 to 16 509]) than in the control group (10 153 [95% CI, 8407 to 13 540]) at 6 months after surgery (*P* < .001). In addition, time spent on vigorous physical activity was significantly higher in the intervention group (34.3 [95% CI, 15.1 to 57.6] minutes) than in the control group (13.7 [95% CI, 7.0 to 33.7] minutes) at 6 months after surgery (*P* < .001; eTable 3 in Supplement 2).

**Figure 2.**
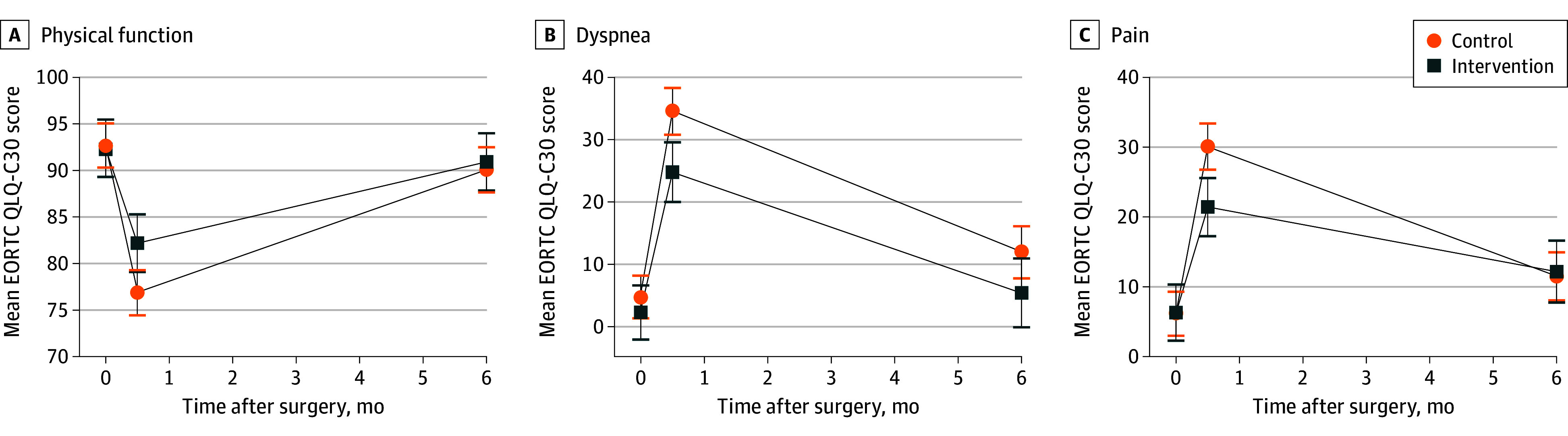
Changes in Patient-Reported Physical Function, Dyspnea, and Pain Scores by Group Scores on the European Organisation for Research and Treatment of Cancer 30-Item Core Quality of Life Questionnaire (EORTC QLQ-C30) for patient-reported physical function (A), dyspnea (B), and pain (C) are reported as means (SDs). Outcomes were measured at baseline (before surgery), 2 weeks after surgery, and 6 months after surgery.

In terms of patient-reported function and symptoms, the intervention group had a smaller decrease in patient-reported physical function scores from baseline (−10.2 [95% CI, −13.9 to −6.5]) compared with the control group (−15.7 [95% CI, −18.6 to −12.9]; Figure 2A) at 2 weeks after surgery. There was a statistically significant difference in patient-reported physical function between the intervention and control groups 2 weeks after surgery, with higher physical function observed in the intervention group (mean [SD] score, 82.2 [17.3] vs 76.9 [17.5]; *P* = .04) (Figure 2A and Table 3).

**Table 3.** Patient-Reported Function, Symptoms, and Quality of Life at Baseline and 2 Weeks and 6 Months After Surgery, by Group^a^

Scale item	Baseline		2 wk After surgery		6 mo After surgery	
	Mean (SD)	*P* value	Mean (SD)	*P* value	Mean (SD)	*P* value
**Function**						
Physical function						
Control	92.7 (12.1)	.83	76.9 (17.5)	.04	90.1 (11.7)	.63
Intervention	92.3 (8.9)		82.2 (17.3)		90.9 (10.7)	
Social functioning						
Control	87.8 (23.1)	.02	85.7 (24.2)	.04	95.7 (13.9)	.09
Intervention	95.0 (18.2)		92.6 (19.7)		98.6 (6.0)	
Role functioning						
Control	97.5 (7.7)	.06	83.3 (28.5)	.13	94.4 (15.4)	.92
Intervention	99.3 (4.3)		89.4 (23.8)		94.1 (18.6)	
Emotional functioning						
Control	80.4 (22.5)	.84	85.1 (20.3)	.70	88 (16.6)	.59
Intervention	81.1 (19.9)		83.9 (22.9)		86.5 (20.9)	
Cognitive functioning						
Control	88.5 (14.9)	.40	93 (13.3)	.43	87 (16.5)	.85
Intervention	90.3 (14.3)		94.4 (8.8)		87.4 (18)	
**Symptoms**						
Dyspnea						
Control	4.7 (11.7)	.12	34.5 (31.6)	.03	12 (23.3)	.01
Intervention	2.3 (8.4)		24.8 (27.1)		5.4 (12.4)	
Pain						
Control	6.1 (12.2)	.92	30.1 (26.8)	.01	11.5 (16.5)	.8
Intervention	6.3 (14.3)		21.4 (20.2)		12.1 (15.5)	
Fatigue						
Control	16.2 (20.9)	.18	33.1 (25.0)	.22	18.8 (20.3)	.51
Intervention	12.5 (14.7)		28.7 (22.6)		20.7 (19.1)	
Nausea and vomiting						
Control	1.1 (4.2)	.72	6.0 (14.8)	.93	3.1 (9.4)	.2
Intervention	0.9 (3.8)		5.9 (11.2)		1.6 (6.9)	
Insomnia						
Control	15.3 (30.2)	.60	27.7 (38.2)	.3	13.7 (30.5)	.13
Intervention	17.6 (28.3)		22.1 (34.6)		20.7 (32.5)	
Appetite loss						
Control	4.4 (16.1)	.57	35.3 (40.0)	.98	7.8 (22.0)	.82
Intervention	5.9 (17.8)		35.1 (37.8)		7.2 (16.8)	
Constipation						
Control	8.3 (21.7)	.18	21.8 (30.2)	.66	5.4 (16.9)	.1
Intervention	4.5 (14.9)		19.8 (32.6)		2.3 (8.5)	
Diarrhea						
Control	3.9 (11.6)	.68	5.4 (18.0)	.48	4.2 (14.8)	.58
Intervention	3.2 (12.5)		3.6 (15.2)		3.2 (11.3)	
**Global health status and quality of life**						
Control	66.7 (20.5)	.16	56.3 (21.7)	.75	69.6 (18.9)	.72
Intervention	70.9 (20.3)		57.3 (18.6)		70.6 (17.8)	

^a^ Scores ranged from 0 to 100, with a higher score indicating more severe symptoms and better functioning and better global health status and quality of life.

Regarding the change in symptoms at 2 weeks after surgery, dyspnea scores increased by 29.8 (95% CI, 25.1 to 34.5) in the control group and by 22.5 (95% CI 16.5 to 28.5) in the intervention group. At the 6-month postoperative visit, dyspnea scores decreased in both groups; however, the intervention group had less dyspnea than the control group (mean [SD] score, 5.4 [12.4] vs 12 [23.3]; *P* = .01) (Figure 2B and Table 3). Similar patterns for pain were observed in both groups (Figure 2C). The intervention group also had less pain than the control group (mean [SD] score, 21.4 [20.2] vs 30.1 [26.8]; *P* = .01; Table 3).

In the PS-matched dataset, the intervention group had significantly higher physical function at 2 weeks after surgery compared with the control group (mean [SD] score, 81.9 [18.5] vs 75.2 [18.2]; *P* = .05). At the 6-month postoperative visit, the intervention group had less dyspnea than the control group (mean [SD] score, 4.7 [11.7] vs 12.3 [24.9]; *P* = .04) (eTable 4 in Supplement 2).

## Discussion

In this study of patients with lung cancer participating in a perioperative exercise intervention, wearable device use in the intervention group was associated with a smaller decrease in number of daily steps and time spent on MVPA at 2 weeks and with a larger increase in daily steps and MVPA at 6 months after surgery from baseline compared with the control group, although there was no difference in improvement in 6MWD at 6 months after surgery. The intervention group had a significant increase in number of daily steps, exceeding 2000 steps both at 2 weeks and 6 months after surgery compared with the control group. Furthermore, the intervention group recorded twice the amount of time spent on vigorous physical activity compared with the control group at 2 weeks and 6 months after surgery. In addition, the intervention group reported higher patient-reported physical function and lower pain than the control group at 2 weeks after surgery, with lower dyspnea both at 2 weeks and 6 months after surgery.

Given that physical activity at 2 weeks after surgery is related to cardiopulmonary function recovery at 6 months,^3^ improvements in physical activity from the early period after surgery may lead to increased physical activity at 6 months. Immediately after surgery, pulmonary function has been reported to dramatically decrease with worse postoperative symptoms, including dyspnea.^2^ Therefore, postoperative exercise should focus on gradually increasing walking duration and distance during rehabilitation. Accordingly, we implemented a goal-setting approach focusing on daily step counting, aiming for a gradual increase in daily steps (eFigure 3 in Supplement 2). Wearable devices were used to provide patients with personalized recommendations for activity intensity, time, frequency, and volume, with data reviewed weekly or biweekly. Two months after surgery, we also updated the program to increase MVPA time. Accordingly, our results showed an increase in step count and MVPA time in the intervention group compared with the control group (usual care) from baseline to 6 months after surgery.

Studies have shown that wearable devices are effective in promoting physical activity.^25,26^ A 2022 systematic review and meta-analysis showed that interventions using these devices resulted in an extra 1800 steps per day and a 6-minute-per-day increase in MVPA in healthy and clinical populations.^26^ Wearable devices are designed to encourage self-monitoring and goal-setting behavior, and patients receive continuous feedback and reminders to stay active and maintain their activity levels. These self-management techniques play a crucial role in improving physical activity by increasing self-efficacy—that is, individuals’ belief in their ability to engage successfully in physical activity.^27^ Increased self-efficacy leads to sustainable behavioral changes that gradually improve daily step count and time spent exercising, encouraging the adoption of healthier habits from the preoperative phase to the postoperative phase. Indeed, our study showed a lower rate of missing wearable device data at only 5%, indicating a relatively higher compliance rate. On the other hand, a previous study to assess the feasibility of physical activity self-monitoring after discharge in patients who had undergone lung cancer surgery showed an acceptable retention rate (41 of 57 [72%]) but a relatively high rate of missing accelerometer data (31%).^28^ Given that patients familiar with wearable devices were more likely to participate in our study and the intervention was conducted with biweekly telephone monitoring, further studies are required to confirm our findings outside the clinical setting.

 No difference in 6MWD was observed in our study. The 6MWT primarily focuses on walking distance and is commonly used to assess functional exercise capacity in patients with lung cancer. However, previous studies have suggested that the 6MWT has a “ceiling effect” in patients with relatively preserved cardiopulmonary function,^29,30^ potentially limiting its effectiveness in evaluating the effect of interventions in these patients. This is because higher cardiorespiratory fitness may result in smaller increments in test performance.^29^ Patients enrolled in the present study were healthier and more physically active than the general population of patients with cancer due to the considerations for lung cancer surgical resection, with a mean initial 6MWD exceeding 500 m. However, given that changes in physical activity over time, such as daily steps and MVPA time, are closely related to maximal oxygen consumption,^31^ our finding that the intervention group had a higher number of daily steps immediately after surgery and continued to increase their daily step count with vigorous physical activity at 6 months after surgery may indicate an improvement in cardiopulmonary function in the intervention group.

Moreover, our results show that the intervention group had improved patient-reported physical function scores for 4 EORTC QLQ-C30 items (self-care, more vigorous activities requiring mobility, strength, and endurance), compared with the control group, which was consistent with a previous study with meta-anlaysis.^32^ By integrating objective measures with subjective assessments,^33^ we gain a thorough understanding of the effect of the intervention on the enhanced recovery of patients’ physical function.

Regarding symptoms, the intervention group reported less pain at 2 weeks after surgery and less dyspnea at 2 weeks and 6 months after surgery compared with the control group. This finding indicates that the intervention program effectively helped patients manage dyspnea and pain, which are the most common causes of discomfort after lung cancer surgery. Regular physical activity can enhance cardiovascular and respiratory function, potentially leading to reduced dyspnea.^34^ Moreover, physical activity and exercise can trigger the release of endorphins and natural pain-relieving chemicals in the body, contributing to decreased pain perception.^35^ In addition, participation in physical activity and adherence to the exercise program may have had a positive effect on patient psychological well-being, such as decreased stress, improved mood, and increased self-esteem, which may contribute to better overall recovery and pain management.^36^

### Limitations

This trial has several limitations. First, the study was conducted at a single center in South Korea, which has distinct sociodemographic and clinical characteristics, including a high proportion of nonsmokers and women; therefore, the results may not be generalizable to other populations in different settings. Second, the possibility of maturation bias cannot be ruled out, as this was a nonrandomized clinical trial using a controlled before-and-after design. However, the control group was selected from a well-designed prospective study with the same protocol as the trial. Third, the 2 wearable devices differed for outcome measurement at 2 weeks after surgery (device A in the intervention group and device B in the control group), although device B was used at both time 1 (baseline) and time 3 (6 months after surgery). Both devices had limitations, but they included a 3-axis accelerometer and a photoplethysmography sensor from the same company.^37,38^

## Conclusions

In this nonrandomized clinical trial, patients who participated in a perioperative exercise program with personalized activity monitoring with a wearable device showed improvement in postoperative physical activity and related patient-reported outcomes after lung cancer surgery compared with those who received standard postoperative care. This finding supports the development of personalized exercise regimens with wearable devices, advocating for their inclusion in comprehensive perioperative rehabilitation programs.

## Notes

Supplement 1.
Trial Protocol and Statistical Analysis Plan

Supplement 2. **eTable 1.** Characteristics at Baseline of Participants Lost to Follow-Up **eTable 2.** Characteristics at Baseline in the Matched Population **eTable 3.** Six-Minute Walking Distance and Physical Activity at Baseline and 2 Weeks and 6 Months After Surgery, by Group in the Matched Dataset **eTable 4.** Patient-Reported Function, Symptoms, and Quality of Life at Baseline and 2 Weeks and 6 Months After Surgery, by Group in the Matched Dataset **eFigure 1.** Study Design **eFigure 2.** Study Flow Diagram **eFigure 3.** Example of a Study Participant’s Physical Activity Data Acquired From a Wearable Device Over the Postoperative Period

Supplement 3.
Data Sharing Statement
