# Supplementary material for: Wearable Device–Based Intervention for Promoting Patient Physical Activity After Lung Cancer Surgery: A Nonrandomized Clinical Trial
Source: JAMA Netw Open. 2024 Sep 20;7(9):e2434180. doi: 10.1001/jamanetworkopen.2024.34180 (PMC11415788; doi:10.1001/jamanetworkopen.2024.34180)
Supplement: Supplement 1. — Trial Protocol and Statistical Analysis Plan [file jamanetwopen-e2434180-s001.pdf]

The protocol of

CATCH-LUNG:

A comprehensive intervention program for patients undergoing lung cancer surgery

CONTENTS

1. Background 1

2. Objectives 1

3. Study design and process 2

4. Participants 3

5. Sample size determination 3

6. Intervention 4

7. Measurement 11

8. Data analysis 13

9. Ethical considerations 13

10. Financing 14

11. References 15

1. Background

Lung cancer is the most prevalent cancer, accounting for 11.6% of all cancer cases and 18.4% of all cancer-related deaths. The number of patients with lung cancer continues to rise.[1] Approximately 85% of primary lung cancers are non-small cell lung cancer (NSCLC), and surgical resection is considered the standard of care, offering the best chance of a cure.[2] However, only 14% of patients with NSCLC undergo surgical intervention. This population is increasingly experiencing long-term survival issues,[3] and the risk of disability associated with this procedure has gained prominence as a crucial patient-focused outcome.[4]

Lung cancer surgery causes further deterioration of the quality of life (QoL), especially in the first 3–6 months after surgery.[5] For many patients, the risk of impaired QoL after surgery is an important consideration when deciding whether to proceed with the procedure. After surgical resection of lung cancer, reduced QoL may explain the functional limitations, decreased exercise capacity, alterations in pulmonary mechanics,[6] and leg muscle weakness observed in patients.[7]

Physical activity (PA) can potentially improve physical function, including cardiorespiratory and muscle function, as well as QoL in patients with cancer.[8] However, many perioperative patients with lung cancer show reduced PA levels. Improving PA and fitness can help alleviate the physical and emotional symptoms experienced during cancer treatment, including surgery.[9] A decrease in PA following surgery can be attributed to many factors such as pain, drowsiness, surgical attachments, reduced quadriceps strength,[10] ventilatory impairment,[11] and dyspnea,[12] all contributing to decreased exercise tolerance.

Unfortunately, few strategies are currently available to enable immediate postoperative recovery and long-term functioning in patients undergoing lung cancer surgery. The effectiveness of multimodal intervention programs for these patients has not yet been adequately researched. Therefore, the aim of this study is to develop a comprehensive perioperative intervention program for patients undergoing lung cancer surgery.

2. Objectives

The aim of this study is to evaluate the development and efficacy of a comprehensive perioperative intervention program to improve postoperative PA in patients with lung cancer. We hypothesized that the perioperative comprehensive intervention program would improve cardiopulmonary function and QoL compared with the historical control group.

The primary outcome is the change in the cardiorespiratory function (6-minute walk test [6MWT]) 6 months after surgery compared with baseline and historical control groups. Secondary outcomes are PA, QoL, fatigue and symptoms at baseline and 2 weeks and 6 months after surgery.

3. Study design and process

3.1. Study design

This is a single center, single-arm study with historical controls of patients undergoing pulmonary resection for NSCLC. The results of the intervention group are compared with those of a historical control group. The recruitment of participants is initiated at least one week prior to surgery. The intervention is carried out continuously from baseline (at the time of recruitment) until six months after surgery.

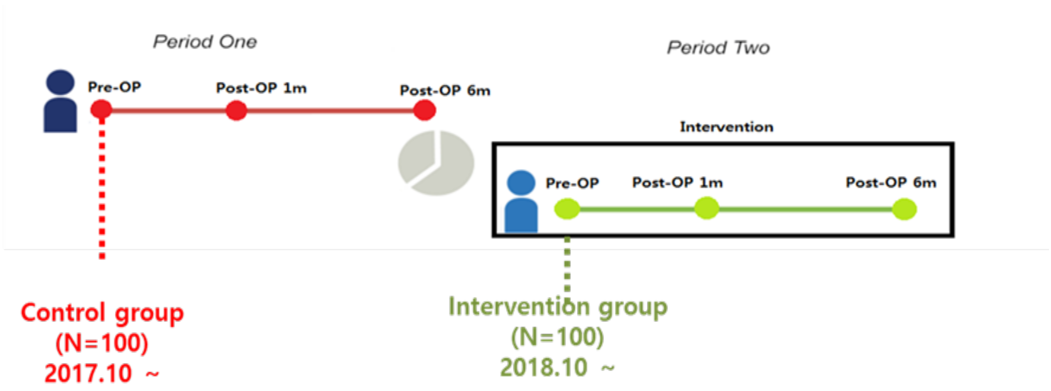

Figure 1. Study design

3.2. Study process

	2018					2019						2020		
	4–5	6–7	8–9	10–11	11–12	1–2	3–4	5–6	7–8	9–10	11–12	1–2	2–3	3–4
Study development	■	■												
Pilot study			■											
Enrollment				■	■	■	■	■						
Allocation and intervention				■	■	■	■	■	■	■	■			
Data coding and clearing					■	■	■	■	■	■	■	■		
Data analysis													■	■
Writing report														■

4. Participants

4.1 Inclusion criteria

1) Patients with confirmed or highly suspected NSCLC;

2) Male or female, aged 18–75 years;

3) Patients with T1-2N0M0 as a clinical TNM stage;

4) Patients who are scheduled for curative pulmonary resection larger than lobectomy;

5) Patients who can walk independently with the Eastern Cooperative Oncology Group Performance Status (ECOG PS) ≤1;

6) Patients who can use a smartphone;

7) Patients who understand the purpose of this study and voluntarily sign the informed consent form.

4.2 Exclusion criteria

1) Patients with multiple primary cancer, metastatic lung cancer, recurrent lung cancer, or history of other cancer in the last 3 years;

2) Patients who have received neoadjuvant chemotherapy and/or radiotherapy;

3) Patients who have not been measured at baseline because of changes in surgery schedule, treatment plan, or withdrawal of consent;

4) Patients who are confirmed as not pathologically NSCLC or metastatic after surgery;

5) Patients with limited physical capacity to participate in this intervention, including those with cardiac failure (New York Heart Association functional classes III and IV)), cerebrovascular disease (cerebral hemorrhage, cerebral infarction, transient ischemic attack, stroke, cerebrovascular accident), gait disability such as osteoarthritis (arthritis, osteoporosis, joint pain, rheumatoid arthritis), and kidney disease (chronic kidney failure).

4.3 Drop-out criteria

1) Need to receive adjuvant chemotherapy and/or radiotherapy;

2) Withdrawal of consent;

3) Researcher’s discretion that it is difficult to continue this trial.

5. Sample size determination

Given that we had co-primary endpoints, our sample size calculation was based on the primary endpoint that necessitated the larger sample size of the two. In this study, we aimed to compare

6-minute walking distance (6MWD) and physical activity at 6 months after surgery between the intervention and control groups, with a mean effect size (Cohen $d = 0.5$) of $\alpha = 0.05$ and 90% power. To achieve this, we needed 85 participants per group. Allowing for 20% loss to follow-up, we needed 100 participants per group.

6. Intervention

6.1. Comprehensive intervention program

1. Conceptual frame work

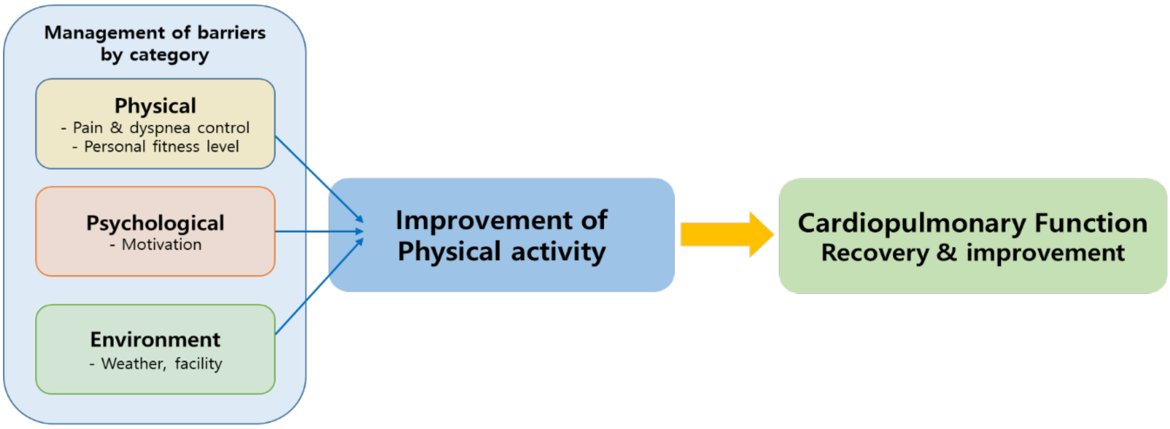

Figure 2. Precede-proceed model for physical activity improvement

2. Time of the intervention: Phases I, II, and III

- Phase I: Preoperative intervention (from diagnosis to surgery).

- Phase II: Immediate postoperative intervention (from discharge to 2 months after surgery).

- Phase III: Postoperative intervention (from 2 months until six months after surgery).

*Outcomes are assessed at baseline (before surgery) and at 1 and 6 months after surgery.

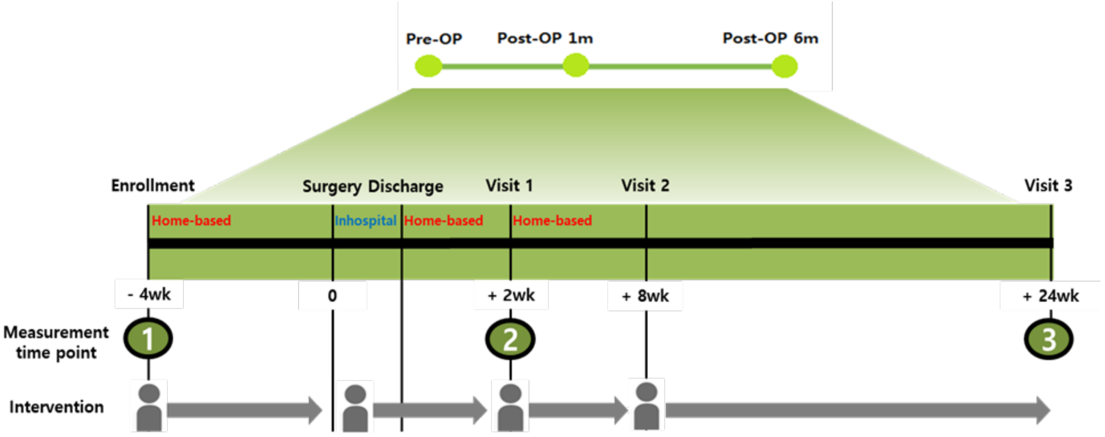

Figure 3. Intervention duration and timing of outcome assessments

6.2. Baseline assessment for Comprehensive intervention program

1. Exercise experience: Check using the International Physical Activity Questionnaire-Short Form (IPAQ-7) and PA (Fitbit): IPAQ-7 moderate-to-vigorous physical activity (MVPA) >150 min/week and Fitbit (Flex): MVPA time > 30 min/day.

2. Comorbidities and cardiac risk: Check using questionnaires and cardiopulmonary function testing (CPET and 6 MWT) for diabetes, hypertension, cardiac failure (New York Heart Association functional classes I and II), lung disease (COPD, asthma, bronchiectasis, chronic bronchitis, emphysema, and interstitial lung disease), and arthritis (walkable).

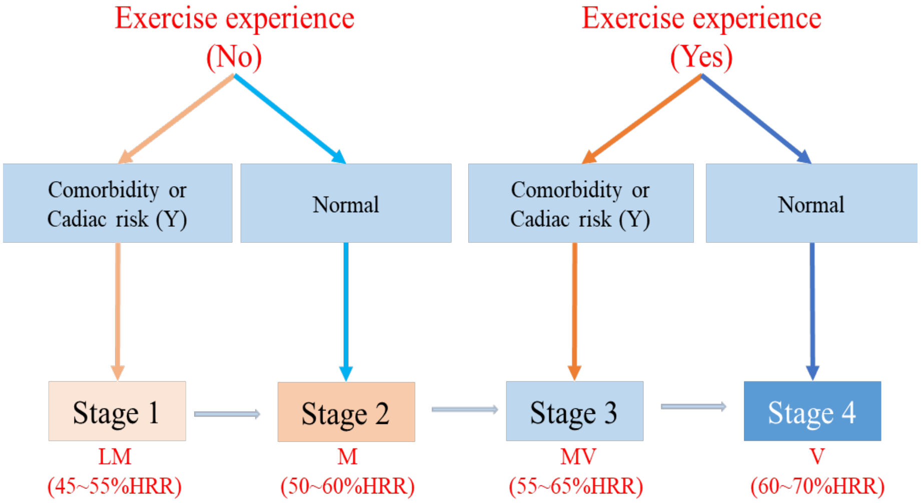

Figure 4. Algorithm for setting exercise intensity levels adjusted for comorbidity and cardiac risk

* Exercise experience (Y, N): IPAQ-7 MVPA ≥150 min/week & Fitbit: MVPA ≥30 min/day
**Comorbidity (Y, N): Diabetes, hypertension, lung disease (COPD), arthritis
***Cardiac risk (Y, N): Arrhythmia, beta blocker, ST-level depress >1 mm during cardiopulmonary exercise test (CPET), Hypertension (DBP >100) during CPET at baseline

For example:

Age	HRmax	HRrest	HRR 60%	HRR 80%
45	220 − 45 = 175	80	(175 − 80)×0.6 = 80 = 137	(175 − 80) × 0.8 = 80 = 156
75	220 − 75 = 145	80	(145 − 80) × 0.6 = 80 = 119	(145 − 80) × 0.8 = 80 = 132

3. Symptoms: Check using pain scale (numeric rating scales), dyspnea, depression

6.3. Component of comprehensive intervention program

In the intervention group, the exercise component consists of 30 min of general education on exercise at baseline. The exercise program is individualized based on the baseline fitness test (according to the American College of Sport Medicine [ACMS] standard). It includes a 5 min warm-up, either 30–90 min of endurance exercise (starting at 45%–70% of heart rate reserve [HRR]), respiratory muscle training using an inspirometer (10 rep. × 3 times per day), and a 5 min cool-down, 5 days per week. If required, additional muscle strength exercises are provided.

All exercises are clearly explained and demonstrated at the beginning of the training. Patients are asked to perform this program at home unsupervised and monitored weekly (bi-weekly) by telephone. A wearable device (Versa, Fitbit Inc.) is used to monitor and record steps taken, heart rate, exercise intensity and duration of activity. In addition, we utilize the real-time activity feedback provided by the wearable device.

At the beginning of the study, we perform a pre-exercise assessment with the CPET to determine the optimal individual exercise dose and identify cardiopulmonary limitations and contraindications. In this web-based approach, the CPET is mandatory to prescribe appropriate exercise and protect participants from overload.

Goal resetting and progression occur when participants complete the goal of the previous week (>70%). Participants receive the WAT, including real-time feedback, a heart rate monitor (to monitor compliance), and a logbook to report the frequency of exercises completed and record all activities (Fig. 4).

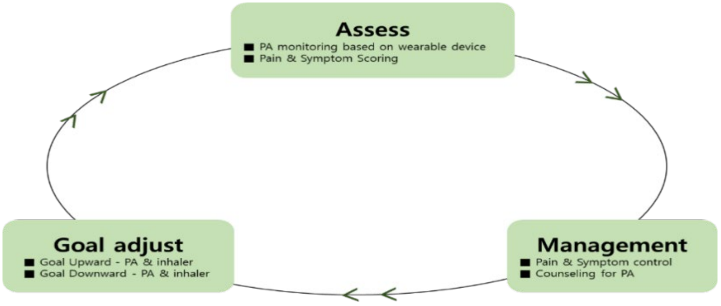

Figure 5. Goal adjustment and management cycle

Table 1. Component of the endurance exercise program by intervention phase

		Phase I	Phase II		Phase III
**Term**		Before surgery (1–6 weeks)	Discharge ~3 weeks after surgery	~2 months after surgery	~6 months after surgery
**Inspirometer**		10 rep. × 3 times/day	10 rep. × 3 times/day	10 rep. × 3 times/day	-
**Endurance exercise**	**Frequency**	5 days per week			
	**Intensity**	%HRR 45–70%[1] (RPE 13–15)	Lower than HR_{peak} during 6MWT (<4METs)	Lower than HR_{peak} during 6MWT (RPE 13–15)	%HRR 45–70% (RPE 13–15)
	**Time**	MVPA≥30 min Baseline MVPA + 20 min/day	≤30 min/1time ≤60 min/day	≤60 min/1time ≤90 min/day	MVPA≥30 min
	**Type**	Brisk walking, climbing, stair climbing, etc.	Walking	Walking, brisk walking	Brisk walking, climbing, stair climbing, etc

Table 2. Progression of goal adjustment by intervention phase

		Phase I	Phase II		Phase III
**Goal achievement**		**MVPA**	**Steps/day**	**Steps/day**	**MVPA**
**Yes (>70%)**	Increase	+ 10–30 min (1 day≥30 min)	+ 1000–3,000 steps/day	+ 1000–3,000 steps/day	+10–30min (≤90 min/day)
	Maintain (30~70%)	Maintain			
**No (≤30%)**	Decrease	− 10–30 min (1 day≥30 min)	− 1,000–3,000 steps/day	− 1,000–3,000 steps/day	− 10–30min (1 day≥30 min)

Goal achievement; time achieve days/5 days * 100%

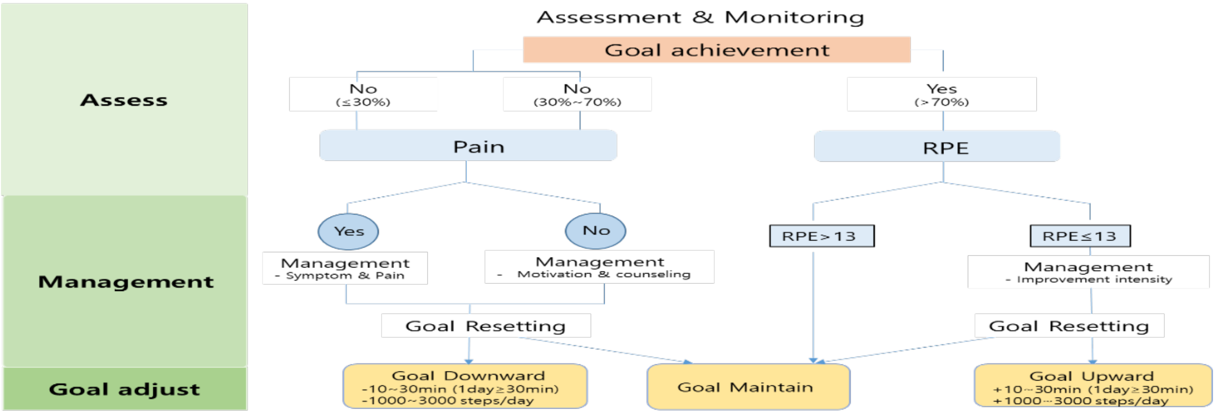

Figure 6. Algorithm for goal adjustment

[1] %HRR45 ~70%

HR_{max} = 220 − age or HR_{peak} of CPET test

%HRR (heart rate reserve %) = Exercise Intensity % × (HR_{max} − HR_{rest}) + HR_{rest}

Table 3. Counseling and monitoring by intervention phase

	Preop period	Early postop period		Late postop period
**Counseling**	1) Face-to-face education<br>2) Tele counseling (biweekly)	1) In hospital education at discharge<br>2) Tele counseling<br>3) Face to face	1) Tele counseling (every 2 weeks)<br>2) Face to face	- Tele counseling (biweekly)
**Monitoring**	Continuous monitoring using WAT	Continuous monitoring using WAT	Continuous monitoring using WAT	Continuous monitoring using WAT

1) Preoperative period (phase 1)

During the preoperative intervention period, all participants receive a one-time personal training session that includes instructions on how to perform aerobic exercise (type, intensity, time and frequency), breathing muscle exercises and the use of the wearable device (Versa, Fitbit, USA). Participants receive printed education materials previously developed "Guidebook for healthy life after lung cancer surgery." The exercise regimen is as follows.

- **Frequency:** 5 days per week
- **Intensity:** HRR 45%–70% (Figure 5)
- **Time:** MVPA≥30 min or baseline MVPA + 20 min/day
- **Type:** walking, climb, stair climbing (if need, weight-bearing exercise)
- **Goal achievement**: time achieve day /5 days
- **Initial setting**: set up the initial goal based on patient baseline PA
- **Progression**: every 2 weeks
- Goal increase (70%): increase MVPA 10–30 min (≤90 min/day)
- Goal achievement (30%–70%): maintain
- Goal fail (30%): decrease MVPA 10–30 min (≥30 min/day)
- **Counseling:** 1) Face-to-face education, 2) Tele counseling (every 2 weeks)
- **Monitoring**: Continuous monitoring using WAT

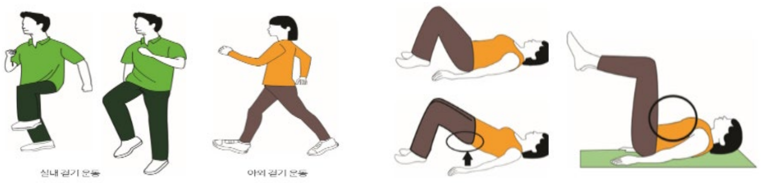

Figure 4. Example of aerobic exercise and muscle strength

2) Early postop period (phase 2)

Immediate postoperative period until discharge.

Patients receive standard postoperative care, including respiratory muscle training and ambulation, in the ward. We recommend ten repetitions with the incentive spirometer three times daily and at least 30 min of ward ambulation three times daily.

Upon discharge, the 6 MWT is conducted on patients in the intervention group to assess their condition and ensure the safety of the exercise regimen. During the 6 MWT, we

monitor oxygen saturation and heart rate (HR). The results, including 6 MWD, maximal HR (HRmax), and minimal oxygen saturation, are recorded and used to tailor the exercise plan. We aim to set the target HRmax during phase 2 to not exceed the HRmax recorded during the 6 MWT. Patients with a history of cardiac problems or those who have experienced postoperative cardiovascular complications undergo continuous EKG monitoring during 6WMT; if there are any indications of cardiac distress or discomfort, a formal consultation with a cardiologist is initiated. Additionally, patients in the intervention group will receive an extra 30 min of education based on their 6 MWT before being discharged.

Until two months postoperative period.

From discharge until one month postoperatively, we recommend engaging in light walking and bedside activities not exceeding 4 METs and maintaining a maximum HR lower than the HRmax recorded during the 6 MWT. Additionally, patients should not exceed an increase of more than 3000 steps beyond the step count recorded on the day before discharge. The exercise regimen is as follows.

- **Frequency:** 5 days per week
- **Intensity:** Lower than HR_{peak} during 6 MWT (<4 METs)
- **Time:** ≤60 min/day
- **Pattern**:
  - discharge until after 1 month: ≤30 min/time
  - until after 2 months: ≤60 min/time, ≤90 min/day
- **Type:** walking & breathing
- **Goal achievement:** Steps achieve day /5 days
  - Initial setting: ≥steps per day at discharge, ≤3000 steps)
- **Progression:** every 1–2 weeks
  - Goal achievement (70%): increase 1000 steps to ~3,000 steps
  - Goal maintain (30%–70%): maintain
  - Goal fail (30%): decrease 1,000–3,000 steps
- **Counseling:** 1) In hospital education at discharge, 2) Tele counseling, 3) Face-to-face
- **Monitoring:** Continuous monitoring using WAT

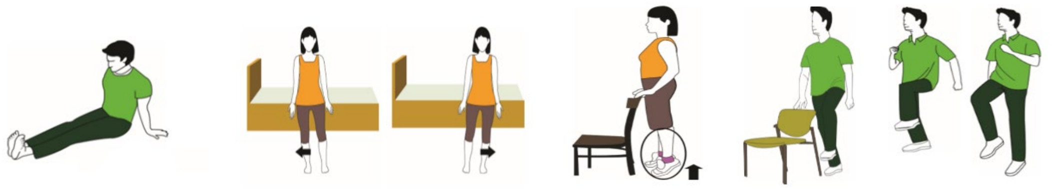

Bed side exercise example

3) Late postoperative period (phase 3)

In the first 2 to 6 months after the operation, the program is adapted to the patients' conditions and the changes in their activities. If cardiovascular complications are observed or suspected immediately after the operation (procedure II), the patient is referred to the circulatory department for further clarification. Upon confirmation of no abnormalities, moderate to high-intensity exercise is recommended as preoperative exercise.

If circulatory problems persist, goal adjustment is made based on steps and time in low-intensity exercise (steps per day), as in Intervention II. Unless specific circumstances exist, the standard exercise goal to enhance cardiopulmonary function is set at 30 min of moderate-intensity exercise. The progress is monitored at 2–3 weeks intervals and goals are adjusted during telephone consultations. The increase in exercise duration is set at 10 minutes and can be increased, decreased, or maintained up to 30 minutes with patient consent. If a target is not met, the cause is investigated to increase PA. The exercise program is as follows.

- **Frequency:** 5 days per week
- **Intensity:** HRR 45%–70% (Figure 5)
- **Time:** MVPA ≥30 min
- **Type:** walking, climb, stair climbing (if need, weight-bearing exercise)
- **Goal achievement**: time achieve day /5 days
- **Initial setting**: set up the initial goal based on patient baseline PA
- **Progression**: biweekly
    - Goal increase (70%): increase MVPA 10–30 min (≤90 min/day)
    - Goal achievement (30%–70%): maintain
    - Goal fail (30%): decrease MVPA 10–30 min (≥30 min/day)
- **Counseling:** 1) In hospital education at discharge, 2) Tele counseling, 3) Face to face
    - **Monitoring:** Continuous monitoring using WAT

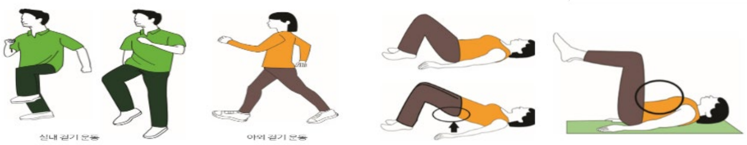

Example of endurance exercise and muscle strength

4) Management for barriers by category

Table 4. Example of barrier management by category

Aim of intervention \ Factor		Physical	Psychological	Environmental
Improvement PA	Lifestyle	- Monitoring physical activity amount and pattern (sedentary activity time) using wearable devices	- Physical activity expert feedback<br>- Self-monitoring with immediate feedback using wearable devices	- Education on indoor exercise methods depending on the season and environment<br>- Providing information on the community exercise environment (use of facilities, etc.)
	Exercise	- Respiratory rehabilitation exercise to improve breathing function<br>- Aerobic (walking) exercise to improve cardiopulmonary function<br>- Strength training to improve activity daily living<br>- Education on type, time, intensity, and frequency of exercise considering the postoperative period through regular phone visits	- Enhancing self-efficacy by suggesting appropriate level goals<br>- Promote exercise practice and maintenance through encouragement and support	

Symptoms management	Physical symptoms	- Screening for postoperative symptoms such as cough, phlegm, difficulty breathing, and pain at the surgical site<br>- Counseling for symptom control<br>- Prevent complications by connecting to medical treatment when necessary	- Mental support through face-to-face counseling and phone visits<br>- Eliminate anxiety by providing prior information about symptoms that may be experienced after surgery	- Forming relationships with experts through regular phone visits<br>- Linkage so that you can see professional medical staff in a timely manner depending on your symptoms
	Mental symptoms	- Counseling after screening for depression, anxiety, sleep disorders, and distress<br>- Prevent complications by connecting to medical treatment when necessary		
Nutrition education		- Nutritional status assessment<br>- Nutritional management for wound recovery after surgery and maintenance of appropriate weight<br>- Nutritional counseling to control body fat and maintain muscle mass related to decreased cardiorespiratory function<br>- Dietary training to overcome loss of appetite		- Simple recipe training according to individual circumstances (e.g., seniors living alone)

7. Measurement

7.1 Definition of outcomes

○ Primary outcome and co-primary outcome

1) Cardiopulmonary function 6 months post-operation: 6-min walking distance (m)

2) Physical activity 6 months post-operation: the number of daily steps

○ Secondary outcome

1) Physical fitness 2 weeks and 6 months post-operation: 6-min walking distance (m), grip strength (kg), and 30-s chair stand

2) Change in the level and intensity of PA at postoperative 2 weeks and 6 months

3) Pulmonary function 2 weeks and 6 months post-operation: FEV_1, FVC, FEV_1/FVC, and DLco

4) The score and each change of symptoms and QoL 2 weeks and 6 months post-operation: dyspnea, pain, fatigue, depression, and QoL

5) Incidence of post-operation complications

7.2. Measurement tool

Variables		Tool	Method
**Outcomes**	Cardiopulmonary function	6-min walk test	Measurement
	Muscle strength	Grip strength test	Measurement
		30-s chair stand test	Measurement
	Pulmonary Function Test	Spirometry test	EMR review
		Diffusing capacity	EMR review
	Physical activity	Wearable activity tracker	Measurement
		International Physical Activity Questionnaire-Short Form (IPAQ-SF)	Questionnaire
	Symptoms and QoL	European Organization for Research and Treatment of Cancer Quality of Life Questionnaire (EORTC QLQ) - core30 (C30) and -lung cancer (LC13)	Questionnaire
		Dyspnea: modified Medical Research Council (mMRC)	Questionnaire
		Dyspnea: COPD Assessment Test (CAT)	Questionnaire
		Fatigue: Brief Fatigue Inventory (BFI)	Questionnaire
		Depression: Patients Health Questionnaire (PHQ9)	Questionnaire
	Complications	All postoperative complications	EMR review
**Confounders**	Body composition	Bioelectrical Impedance Analysis (BIA)	Measurement
	Health behaviors	Mini Dietary Assessment (MDA)	Questionnaire
		Smoking and drinking status	Questionnaire
	Cognitive function	Mini Mental State Examination (MMSE)	Questionnaire
	Socio-demographic factors	Age, sex, marital status, educational level, income	Questionnaire
	Clinical factors	Comorbidity, stage, surgery type, adjuvant treatment, blood test	EMR review

7.3. Measurement time

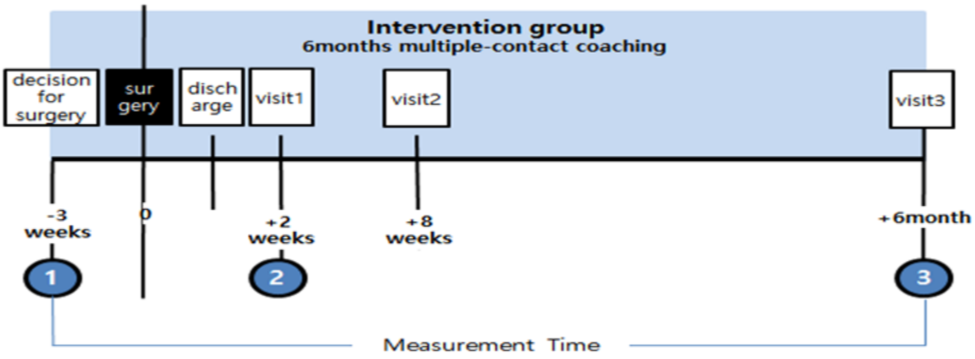

8. Data analysis

We use an intention-to-treat approach for all analyses. The primary and co-primary outcomes were cardiopulmonary function and physical activity at 6 months after surgery, measured by the 6-minute walking distance (6MWD) and the number of daily steps. Physical activity was also measured with the time spent of MVPA as secondary outcome. Secondary outcomes were change in physical activity (number of daily steps and time spent of MVPA) at 2 weeks after surgery, physical activity (time spent of MVPA) at 6 months after surgery and changes in health related QOL including function and symptoms at 2 weeks and 6 months after surgery, using linear regression model.

Outcomes were compared using linear regression and a mixed model at each time point and change, respectively. For outcomes measured at 3 visits, we used linear mixed-effects models with the main effects of visits and visit group interaction terms to assess differences in outcomes when comparing the intervention and control groups. Random intercept effects were included to account for differences in outcomes between study participants at baseline. Statistical significance is set at P-value <0.05 as statistically significant. All analyses are performed using STATA version 16 (StataCorp LP, College Station, TX, USA).

9. Ethical considerations

9.1 Protection of participants

This study is conducted in accordance with the ethical principles that have their origin in the Declaration of Helsinki and that are consistent with Good Clinical Practice (GCP) and the applicable regulatory requirement(s).

9.2 Informed consent

All participants and their legally acceptable representatives are informed of the purpose of the study.

We state that this study is conducted only for participants who have voluntarily confirmed their willingness to participate, and that they may refuse to participate in or withdraw from the study at any time without penalty or loss of benefits to which they are otherwise entitled. We also explain that signing the written informed consent form and participating in this study does not constitute a waiver of any of the participant's legal rights.

Informed consent is obtained on written, signed and dated forms. A copy of the signed informed consent form is given to participants and the original signed informed consent form is retained by the investigator.

9.3 Approval of Institutional Review Board

This study is approved by the Institutional Review Board (IRB) of Samsung Medical Center in Seoul, Korea (IRB no. SMC2015-11-025) to protect the rights, safety, and well-being of the participants.

9.4 Registration of clinical trial

Clinical Trials.gov ID: NCT03215537

9.5 Confidentiality

The records identifying the participants are kept confidential and, to the extent permitted by the applicable laws and/or regulations, they are not made publicly available. The confidentiality of the participant's information is assured and participant's identity is not disclosed to the public. This information is only used for statistical analysis for the purposes of this study. If the study results are published, the participants' identities remain confidential.

Participant identification records are coded with a unique identification number assigned by the examiner to protect their confidentiality.

During the collection, processing and storage of the information in the study, we use a security system to prevent unauthorized access to the data.

10. Financing

This work is supported by the National Research Foundation of Korea (NRF) grant funded by the Korean government (MSIP) [Nos. 2015R1C1A2A01055805 and 2017R1A2B2006435].

11. References

1. Bray, F., et al., *Global cancer statistics 2018: GLOBOCAN estimates of incidence and mortality worldwide for 36 cancers in 185 countries.* CA: A Cancer Journal for Clinicians, 2018. **68**(6): p. 394-424.
2. Aokage, K., et al., *Limited resection for early-stage non-small cell lung cancer as function-preserving radical surgery: a review.* Japanese Journal of Clinical Oncology, 2017. **47**(1): p. 7-11.
3. Cykert, S., *Risk acceptance and risk aversion: patients' perspectives on lung surgery.* Thoracic Surgery Clinics, 2004. **14**(3): p. 287-293.
4. Cykert, S., *Risk acceptance and risk aversion: patients' perspectives on lung surgery.* Thorac Surg Clin, 2004. **14**(3): p. 287-93.
5. Win, T., et al., *Effect of lung cancer surgery on quality of life.* Thorax, 2005. **60**(3): p. 234-8.
6. Markos, J., et al., *Preoperative assessment as a predictor of mortality and morbidity after lung resection.* Am Rev Respir Dis, 1989. **139**(4): p. 902-10.
7. Nishimura, H., et al., *Cardiopulmonary function after pulmonary lobectomy in patients with lung cancer.* Ann Thorac Surg, 1993. **55**(6): p. 1477-84.
8. Speck, R.M., et al., *An update of controlled physical activity trials in cancer survivors: a systematic review and meta-analysis.* J Cancer Surviv, 2010. **4**(2): p. 87-100.
9. Canario, A.C., et al., *Physical activity, fatigue and quality of life in breast cancer patients.* Rev Assoc Med Bras (1992), 2016. **62**(1): p. 38-44.
10. Arbane, G., et al., *Evaluation of an early exercise intervention after thoracotomy for non-small cell lung cancer (NSCLC), effects on quality of life, muscle strength and exercise tolerance: Randomised controlled trial.* Lung Cancer, 2011. **71**(2): p. 229-234.
11. Larsen, K.R., et al., *Cardiopulmonary function at rest and during exercise after resection for bronchial carcinoma.* Ann Thorac Surg, 1997. **64**(4): p. 960-4.
12. Bolliger, C.T., et al., *Pulmonary function and exercise capacity after lung resection.* Eur Respir J, 1996. **9**(3): p. 415-21.
