# Supplementary material for: Wearable Device–Based Intervention for Promoting Patient Physical Activity After Lung Cancer Surgery: A Nonrandomized Clinical Trial
Source: JAMA Netw Open. 2024 Sep 20;7(9):e2434180. doi: 10.1001/jamanetworkopen.2024.34180 (PMC11415788; doi:10.1001/jamanetworkopen.2024.34180)
Supplement: Supplement 2. — eTable 1. Characteristics at Baseline of Participants Lost to Follow-Up eTable 2. Characteristics at Baseline in the Matched Population eTable 3. Six-Minute Walking Distance and Physical Activity at Baseline and 2 Weeks and 6 Months After Surgery, by Group in the Matched Dataset eTable 4. Patient-Reported Function, Symptoms, and Quality of Life at Baseline and 2 Weeks and 6 Months After Surgery, by Group in the Matched Dataset eFigure 1. Study Design eFigure 2. Study Flow Diagram eFigure 3. Example of a Study Participant’s Physical Activity Data Acquired From a Wearable Device Over the Postoperative Period [file jamanetwopen-e2434180-s002.pdf]

Supplementary Online Content

Lee J, Kong S, Shin S, et al. Wearable device–based intervention for promoting patient physical activity after lung cancer surgery: a nonrandomized clinical trial. *JAMA Netw Open*. 2024;7(9):e2434180. doi:10.1001/jamanetworkopen.2024.34180

eTable 1. Characteristics at Baseline of Participants Lost to Follow-Up

eTable 2. Characteristics at Baseline in the Matched Population

eTable 3. Six-Minute Walking Distance and Physical Activity at Baseline and 2 Weeks and 6 Months After Surgery, by Group in the Matched Dataset

eTable 4. Patient-Reported Function, Symptoms, and Quality of Life at Baseline and 2 Weeks and 6 Months After Surgery, by Group in the Matched Dataset

eFigure 1. Study Design

eFigure 2. Study Flow Diagram

eFigure 3. Example of a Study Participant's Physical Activity Data Acquired From a Wearable Device Over the Postoperative Period

This supplementary material has been provided by the authors to give readers additional information about their work.

© 2024 Lee J et al. *JAMA Network Open.*

eTable 1. Characteristics at Baseline of Participants Lost to Follow-Up[a]

Characteristics	Overall (N=194)	F/U loss (N=65)	p-value
Age, mean (SD), y	60.3 (8.7)	61.2 (7.3)	0.45
Sex			0.01
Male	96 (49.5)	44 (67.7)	
Female	98 (50.5)	21 (32.3)	
BMI, mean (SD)	24.1 (3.0)	24.0 (2.8)	0.72
Body fat, mean (SD), %	27.9 (7.4)	26.3 (7.5)	0.14
Muscle mass, mean (SD), kg	25.4 (5.4)	26.9 (5.1)	0.06
Married	159 (82)	53 (81.5)	0.37
Education			0.006
Less than high school	44 (22.7)	25 (38.5)	
High school or higher	150 (77.3)	37 (56.9)	
Employment status			0.91
Unemployed	83 (42.8)	26 (40.0)	
Employed	111 (57.2)	36 (55.4)	
Income (million, Korean won)	400 (260-600)	400 (200-700)	0.76
Smoking status			<.001
Never	103 (53.1)	22 (33.8)	
Past	69 (35.6)	18 (27.7)	
Current	22 (11.3)	22 (33.8)	
Religious affiliation			0.64
Yes	78 (40.2)	27 (41.5)	
No	116 (59.8)	35 (53.8)	
Comorbidity			
Cardiac	16 (8.2)	8 (12.3)	0.33
Pulmonary	32 (16.5)	18 (27.7)	0.05
Thrombosis	3 (1.5)	4 (6.2)	0.07
Hypertension	57 (29.4)	19 (29.2)	0.98
Diabetes	21 (10.8)	12 (18.5)	0.11
Cerebrovascular accident	3 (1.5)	3 (4.6)	0.17
Lung function			
FVC (L)	3.4 (3-4.3)	3.7 (3.1-4.3)	0.24
FVC (%)	95 (88-105)	92.5 (83-100)	0.09
FEV_1 (L)	2.6 (2.3-3.1)	2.7 (2.3-3)	0.94
FEV_1 (%)	94 (87-103)	88.5 (79-98)	0.009
FEV_1/FVC (%)	76 (71-79)	74 (69-78)	0.07

Abbreviations: BMI, body mass index (calculated as weight 1 in kilograms divided by height in meters squared); FEV_1, forced expiratory volume in 1 second; FVC, forced vital capacity.

[a] Unless indicated otherwise, values are presented as No. (%) of patients.

© 2024 Lee J et al. *JAMA Network Open.*

eTable 2. Characteristics at Baseline[a] in the Matched Population

Characteristics	Control group (N=57)	Intervention group (N=57)	p-value
Age, mean (SD), y	58.9 (8.8)	60.9 (9.3)	0.25
Sex			0.85
Male	28 (49.1)	30 (52.6)	
Female	29 (50.9)	27 (47.4)	
BMI, mean (SD)	24.9 (3.2)	23.7 (3.2)	0.06
Body fat, mean (SD), %	29.1 (7.9)	27.4 (6.8)	0.21
Muscle mass, mean (SD), kg	25.4 (5.2)	25.4 (5.6)	0.98
Married	48 (84.2)	45 (78.9)	0.63
Education			>0.99
Less than high school	13 (22.8)	12 (21.1)	
High school or higher	44 (77.2)	45 (78.9)	
Employment status			0.34
Unemployed	30 (52.6)	36 (63.2)	
Employed	27 (47.4)	21 (36.8)	
Income (million, Korean won)	407.6 (279.5)	437.1 (268.6)	0.58
Smoking status			0.67
Never	28 (49.1)	29 (50.9)	
Past	21 (36.8)	23 (40.4)	
Current	8 (14.0)	5 (8.8)	
Religious affiliation			0.85
Yes	36 (63.2)	34 (59.6)	
No	21 (36.8)	23 (40.4)	
Season at enrollment[b]			0.82
Spring	17 (29.8)	16 (28.1)	
Autumn	15 (26.3)	18 (31.6)	
Winter	25 (43.9)	23 (40.4)	
Comorbidity			
Cardiac	8 (14.0)	3 (5.3)	0.20
Pulmonary	10 (17.5)	8 (14.0)	0.80
Thrombosis	0	0	0.71
Hypertension	18 (31.6)	18 (31.6)	>0.99
Diabetes	5 (8.8)	3 (5.3)	
Cerebrovascular accident	0	0	>0.99
Pulmonary function test			
FVC (L)	3.6 (3.0, 4.4)	3.5 (3.0, 4.2)	0.96
FVC (%)	96.0 (88.0, 106.0)	96.0 (88.0, 105.0)	0.74
FEV_1 (L)	2.7 (2.3, 3.1)	2.6 (2.3, 3.1)	0.66
FEV_1 (%)	94.0 (87.0, 103.0)	91.0 (87.0, 103.0)	0.65
FEV_1/FVC (%)	76.0 (72.0, 79.0)	74.0 (70.0, 79.0)	0.65

Abbreviations: BMI, body mass index (calculated as weight 1 in kilograms divided by height in meters squared); FEV_1, forced expiratory volume in 1 second; FVC, forced vital capacity.

[a] Unless indicated otherwise, values are presented as No. (%) of patients.

[b] Spring: March to May; Summer: June to August, Autumn: September to November; Winter: December to February

© 2024 Lee J et al. *JAMA Network Open.*

eTable 3. Six-Minute Walking Distance and Physical Activity at Baseline and 2 Weeks and 6 Months After Surgery, by Group in the Matched Dataset

Group	Baseline		2 wk after surgery		6 mo after surgery	
	Mean (95% CI)	P-value	Mean (95% CI)	P-value	Mean (95% CI)	P-value
**6-min walking distance, m**						
Control	510.0 (450.0, 576.0)	0.11	430.0 (377.2, 510.0)	0.10	510.0 (480.0, 540.0)	0.02
Intervention	538.0 (510.0, 570.0)		466.0 (422.0, 510.0)		536.0 (495.0, 571.0)	
**No. of daily steps**						
Control	8961 (6602, 12453)	0.73	4193 (3440, 6695)	<0.01	10153 (8407, 13540)	<0.01
Intervention	9452 (6309, 11266)		8346 (6749, 11474)		12717 (9582, 16509)	
**Level of physical activity, min**						
Vigorous						
Control	16.9 (5.0, 33.2)	0.78	3.4 (0.1, 9.9)	<0.01	13.7 (7.0, 33.7)	<0.01
Intervention	17.1 (5.1, 35.1)		20.3 (6.5, 42.8)		34.3 (15.1, 57.6)	
Moderate						
Control	15.7 (9.0, 30.2)	0.90	7.1 (2.1, 14.6)	<0.01	22.2 (13.4, 41.9)	0.49
Intervention	17.1 (8.4, 27.6)		22.0 (10.3, 29.7)		26.3 (15.7, 36.8)	
Light						
Control	239.0 (177.2, 316.0)	0.74	127.0 (77.1, 172.7)	<0.01	255.7 (207.9, 320.0)	0.36
Intervention	231.2 (167.8, 287.5)		160.8 (130.1, 206.2)		236.0 (181.9, 296.8)	

© 2024 Lee J et al. *JAMA Network Open.*

eTable 4. Patient-Reported Function, Symptoms, and Quality of Life at Baseline and 2 Weeks and 6 Months After Surgery, by Group in the Matched Dataset[a]

	Baseline		2 wk after surgery		6 mo after surgery	
	Mean (SD)	P-value	Mean (SD)	P-value	surgery Mean (SD)	P-value
**Function**						
Physical function						
Control	92.8 (12.3)	0.81	75.2 (18.2)	0.05	89.8 (10.8)	0.40
Intervention	92.3 (9.3)		81.9 (18.5)		91.5 (10.0)	
Social functioning						
Control	92.4 (20.4)	0.54	86.0 (26.0)	0.15	94.6 (16.7)	0.07
Intervention	94.7 (19.7)		92.4 (20.9)		98.8 (5.3)	
Role functioning						
Control	98.0 (6.4)	0.27	80.7 (28.7)	0.08	95.3 (12.9)	0.92
Intervention	99.1 (4.9)		89.5 (24.3)		95.0 (16.4)	
Emotional functioning						
Control	81.0 (22.9)	0.38	85.7 (23.4)	0.96	89.9 (13.2)	0.54
Intervention	84.4 (17.9)		86.0 (19.4)		88.0 (19.5)	
Cognitive functioning						
Control	89.2 (13.5)	0.74	94.4 (12.7)	0.66	86.3 (17.3)	0.55
Intervention	90.1 (14.7)		95.3 (8.2)		88.1 (15.1)	
**Symptoms**						
Dyspnea						
Control	4.7 (11.7)	0.11	33.9 (31.2)	0.09	12.3 (24.9)	0.04
Intervention	1.8 (7.5)		24.6 (27.1)		4.7 (11.7)	
Pain						
Control	5.6 (8.5)	0.89	33.6 (27.9)	0.01	12.9 (19.7)	0.44
Intervention	5.8 (13.9)		22.2 (19.8)		10.4 (13.3)	
Fatigue						
Control	14.4 (21.2)	0.66	33.1 (23.7)	0.30	19.4 (19.3)	0.75
Intervention	12.9 (16.1)		28.7 (22.7)		18.3 (16.3)	
Nausea & vomiting						
Control	0.9 (3.8)	>0.99	7.3 (17.8)	0.76	3.2 (10.2)	0.06
Intervention	0.9 (3.8)		6.4 (12.1)		0.6 (3.1)	
Insomnia						
Control	19.9 (34.4)	0.35	36.8 (42.1)	0.03	18.7 (34.5)	>0.99
Intervention	14.6 (25.2)		21.1 (33.7)		18.7 (30.2)	
Appetite loss						
Control	6.4 (21.3)	0.87	41.1 (42.6)	0.39	7.6 (21.8)	0.82
Intervention	5.8 (18.0)		34.5 (37.8)		7.2 (16.8)	
Constipation						
Control	7.0 (18.6)	0.27	28.7 (35.9)	0.06	5.8 (16.8)	0.17
Intervention	3.5 (15.0)		17.0 (30.3)		2.4 (8.7)	
Diarrhea						
Control	4.7 (13.3)	0.30	7.7 (22.9)	0.42	6.4 (19.4)	0.23

© 2024 Lee J et al. *JAMA Network Open.*

Intervention	2.3 (10.7)		4.7 (17.2)		3.0 (9.6)	
Global health status and quality of life						
Control	65.9 (21.6)	0.23	53.5 (21.6)	0.36	70.1 (17.8)	0.77
Intervention	70.6 (20.3)		57.1 (19.9)		71.1 (19.7)	

[a]Scores ranged from 0 to 100, with a higher score indicating more severe symptoms and better functioning and better global health status and quality of life.

© 2024 Lee J et al. *JAMA Network Open.*

eFigure 1. Study Design

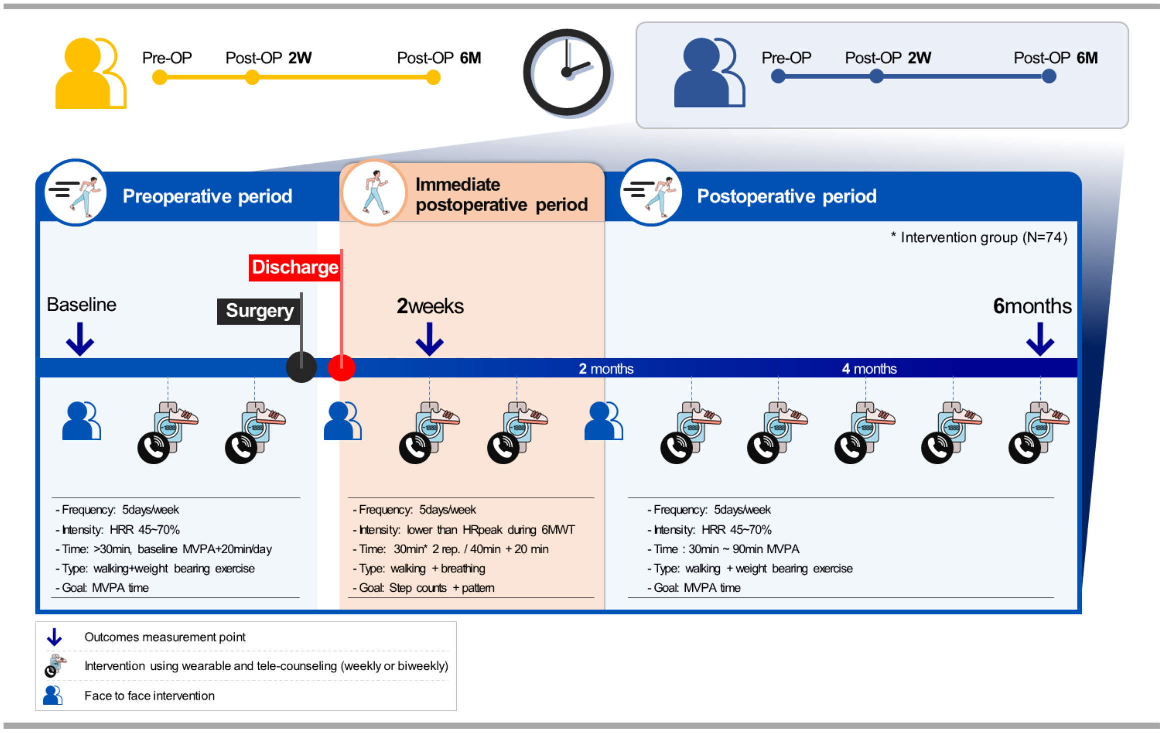

© 2024 Lee J et al. *JAMA Network Open.*

eFigure 2. Study Flow Diagram

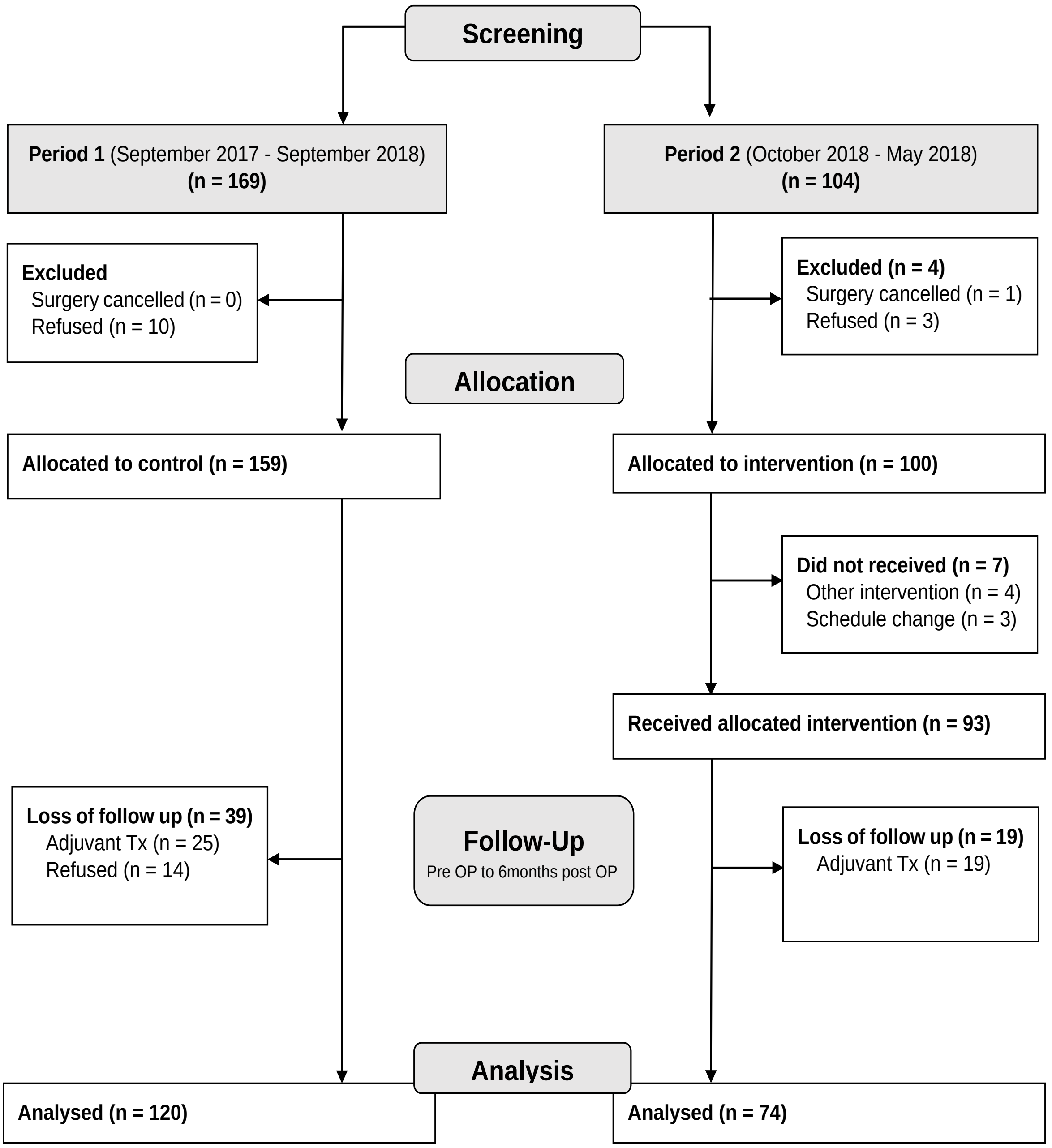

© 2024 Lee J et al. *JAMA Network Open.*

eFigure 3. Example of a Study Participant's Physical Activity Data Acquired From a Wearable Device Over the Postoperative Period

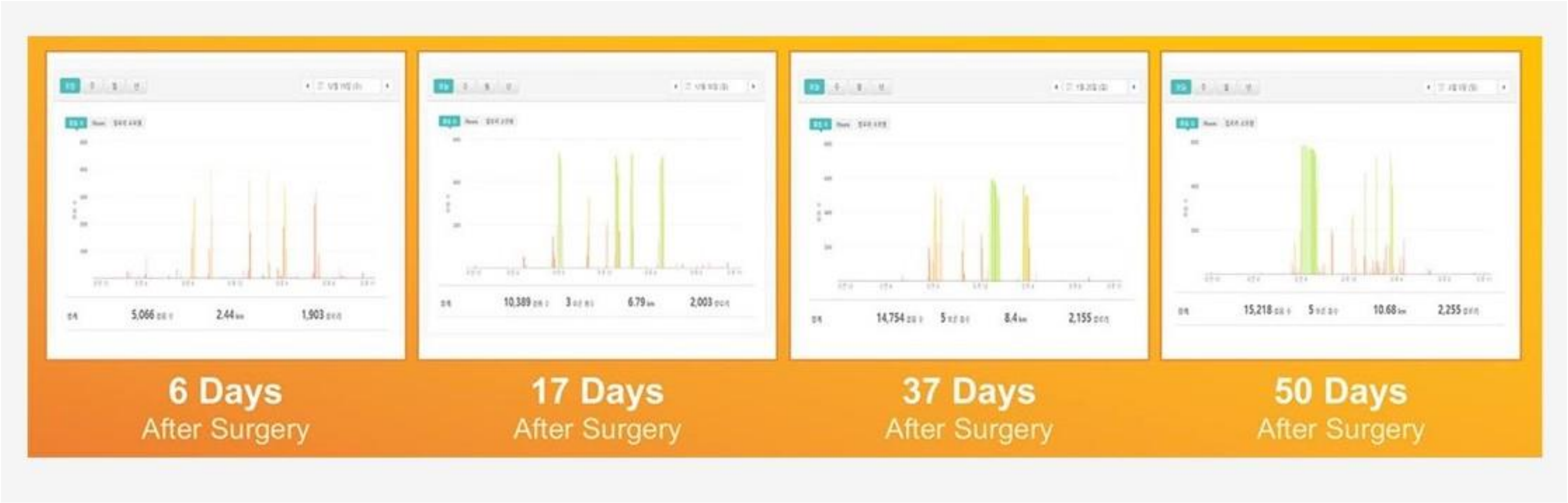

© 2024 Lee J et al. *JAMA Network Open.*
