# Supplementary material for: Wearable Device–Based Intervention for Promoting Patient Physical Activity After Lung Cancer Surgery: A Nonrandomized Clinical Trial
Source: JAMA Netw Open. 2024 Sep 20;7(9):e2434180. doi: 10.1001/jamanetworkopen.2024.34180 (PMC11415788; doi:10.1001/jamanetworkopen.2024.34180)
Supplement: Supplement 3. — Data Sharing Statement [file jamanetwopen-e2434180-s003.pdf]

Data Sharing Statement

Lee. Wearable Device–Based Intervention for Promoting Patient Physical Activity After Lung Cancer Surgery. *JAMA Netw Open*. Published September 20, 2024. doi:10.1001/jamanetworkopen.2024.34180

Data
Data available: No

Additional Information
Explanation for why data not available: The datasets used and/or analyzed during the current study are not publicly available due to patient privacy concerns. However, they may be made available upon reasonable request to the corresponding authors, Dr. Danbee Kang (dbee.kang@gmail.com) and Hye Yun Park (hyeyunpark@skku.edu).
